# Accuracy of BRCA1/2 Mutation Prediction Models for Different Ethnicities and Genders: Experience in a Southern Chinese Cohort

**DOI:** 10.1007/s00268-011-1406-y

**Published:** 2012-01-31

**Authors:** Ava Kwong, Connie H. N. Wong, Dacita T. K. Suen, Michael Co, Allison W. Kurian, Dee W. West, James M. Ford

**Affiliations:** 1 Division of Breast Surgery, Department of Surgery, The University of Hong Kong, Hong Kong, China; 2 The Hong Kong Hereditary Breast Cancer Family Registry, Hong Kong, China; 3 Comprehensive Oncology Cancer Centre, Cancer Genetics Centre, Hong Kong Sanatorium and Hospital, Hong Kong, China; 4 Department of Oncology, Stanford University School of Medicine, Stanford, CA USA; 5 Department of Health Research Policy, Stanford University School of Medicine, Stanford, CA USA; 6 Department of Medicine, Stanford University School of Medicine, Stanford, CA USA

## Abstract

**Background:**

BRCA1/2 mutation prediction models (BRCAPRO, Myriad II, Couch, Shattuck-Eidens, BOADICEA) are well established in western cohorts to estimate the probability of BRCA1/2 mutations. Results are conflicting in Asian populations. Most studies did not account for gender-specific prediction. We evaluated the performance of these models in a Chinese cohort, including males, before BRCA1/2 mutation testing.

**Methods:**

The five risk models were used to calculate the probability of BRCA mutations in probands with breast and ovarian cancers; 267 were non-BRCA mutation carriers (247 females and 20 males) and 43 were BRCA mutation carriers (38 females and 5 males).

**Results:**

Mean BRCA prediction scores for all models were statistically better for carriers than noncarriers for females but not for males. BRCAPRO overestimated the numbers of female BRCA1/2 mutation carriers at thresholds ≥20% but underestimated if <20%. BRCAPRO and BOADICEA underestimated the number of male BRCA1/2 mutation carriers whilst Myriad II underestimated the number of both male and female carriers. In females, BRCAPRO showed similar discrimination, as measured by the area under the receiver operator characteristic curve (AUC) for BRCA1/2 combined mutation prediction to BOADICEA, but performed better than BOADICEA in BRCA1 mutation prediction (AUC 93% vs. 87%). BOADICEA had the best discrimination for BRCA1/2 combined mutation prediction (AUC 87%) in males.

**Conclusions:**

The variation in model performance underscores the need for research on larger Asian cohorts as prediction models, and the possible need for customizing these models for different ethnic groups and genders.

## Introduction

The identification of BRCA1 and BRCA2 mutations has dramatically changed the landscape of breast cancer in the past decade. Testing of these genes has become an important part of clinical practice. Mutations in either of these genes results in increased risk of breast and ovarian cancer, accounting for 5% to 10% of breast cancers and 10% to 15% of ovarian cancers [1–4]. BRCA1 is mutated more frequently in families with both breast and ovarian cancer [5, 6] and more rarely in families with male breast cancer where BRCA2 is predominant [7, 8].

Genetic testing, however, is expensive and may be associated with adverse psychological effects not only to the patient but also family members [9, 10]. Family history of breast cancer is not uncommon, but BRCA mutations are relatively rare. Establishing an efficient way to identify a “high-risk group” accurately for genetic testing is important for patient care. However, the prevalence of germline BRCA mutations in these “high-risk families” is estimated at 13–19% [11, 12]. These low figures lead to the development of models (such as BOADICEA [13], BRCAPRO [14–16], Myriad [11], Couch (also known as PENN) [17], Shattuck-Eidens [18], and Manchester [19]) that can assess the pre-test probability of identifying a BRCA1 or BRCA2 mutation and enable efficient targeting of genetic testing.

Although these models were built by using data from Caucasian populations, they are being used in clinical practice to assess the risk of BRCA1/2 mutation carriage in patients of other ethnic backgrounds [20–22] and have had variable accuracy for African Americans and Hispanics in the United States in different studies [20–23]. Indeed, the chance of carrying a genetic mutation varies between different races and is most common in Ashkenazi Jewish cohorts [24]. For ethnic populations where limited genetic studies have been undertaken, variants of unknown significance detected may be benign mutation changes but some may in fact be characterized to be pathogenic when analyzed further at an RNA level [25]. Hence, evaluation of the performance of these models in different ethnic groups has been performed so that individuals who are at risk can be accurately identified and be offered intensive surveillance and preventative measures.

Although still less-tested than in Caucasian populations, increasing numbers of Asian cohorts are being clinically tested both in Asia and in western countries, such as the United States [26–30]. A few studies have evaluated these models in a mixture of different Asian or Chinese cohorts [31–33]. Most of these studies found an underprediction of BRCA2 mutations with a comparable discriminative ability as for Caucasians. Moreover, none of the studies separately analyzed the accuracy of the use of these models in prediction of mutation carriage for males [32, 34], and therefore, there is still limited reporting of gender-specific prediction.

The purpose of our study was to compare the four commonly used BRCA1/2 mutation prediction models (BOADICEA, BRCAPRO, Myriad II, Couch and Shattuck-Eidens) to determine the likelihood of finding a BRCA1, BRCA2, or combined BRCA1/2 gene mutation in patients residing in Hong Kong, who are mainly Southern China origin Chinese, and to determine if these models can perform accurately for males.

## Materials and methods

### Study population

Participants were recruited through the prospective database at The Hong Kong Hereditary and High Risk Breast Cancer Family Registry (www.asiabreastregistry.com), which was established in March 2007. Protocols of the study were approved by Institutional Review Board of the participating research centers. The Registry collects data from high-risk probands and families referred to the Hong Kong Hereditary and High-Risk Breast Cancer Programme for consideration of genetic testing. Female breast and ovarian cancer patients were accrued based on age of onset, family history suggestive of hereditary predisposition, bilateral breast cancer status, and male breast cancer patients and were recruited from public and private hospitals and centers covering all areas of Hong Kong. Additional details of accrual were published in a previous study [29]. An epidemiological questionnaire, pedigree information about breast, ovarian, and/or other cancers of the first-, second-, and third-degree relatives of each proband was obtained. Unknown ages and unknown year at death were assumed to be 25 years between each generation [35].

The CancerGene software program (CaGene 4.3, The University of Texas Southwestern Medical Center, Dallas, TX) was used to calculate the probability of BRCA1, BRCA2, and BRCA1/2 mutation carriage from BRCAPRO, Myriad II, and Couch and Shattuck-Eidens models. BOADICEA risk model was calculated through https://pluto.srl.cam.ac.uk/cgi-bin/bd1/v1/bd.cgi using the most updated software available. Couch and Shattuck-Eidens models were only calculated in female participants.

All calculations were performed for male and female patients separately to evaluate how accurately BRCAPRO, Myriad II, Couch and Shattuck-Eidens, and BOADICEA models predicted risks for male and female independently. Independent *t* tests were used to compare any difference in mean score computed by the five carrier prediction algorithms between patients with and without BRCA mutation. Pearson χ^2^ goodness of fit test was used to compare the number of mutations predicted by these risk models with the actual number of mutation detected.

The area under the receiver operating characteristic (ROC) curve with 95% confidence interval for each model was used to determine discrimination. ROC was evaluated to compare the ability of these models to distinguish between patients with and without a mutation and to measure the overall performance of each model. The closer the area under the ROC curve (AUC) is to 1, the better the overall performance of the model. A model with an AUC value of 1 is one that is perfectly accurate, whereas an AUC of 0.5 indicates no discriminating ability. The reference line distinguishes subjects who carry the BRCA mutation versus those who do not by pure chance. The resulting ROC curve would fall along this diagonal line, which is referred to as the chance diagonal. The empirical estimates of the sensitivity and specificity for positive BRCA status were calculated at the conventional testing thresholds of 10 and 20 for BRCAPRO, Myriad II, and BOADICEA models.

Fisher’s exact test was used to test for significance for small sample sizes. All tests were two-sided, and *p* values < 0.05 were considered statistically significant difference. All statistical analyses were performed with the SPSS for Windows Release 16.0 (SPSS Inc., Chicago, IL).

## Results

A total of 310 probands (285 females and 25 males) were recruited. All were of Chinese ancestry. Among them, 267 (86.1%) individuals (247 females and 20 males) were noncarriers and 43 (13.9%) individuals (38 females and 5 males) were mutation carriers. Among 285 female probands, most were breast cancer patients (98%), and 247 (86.7%), 15 (5.3%), and 23 (8.1%) were noncarriers, BRCA1, and BRCA2 carriers respectively. Among 25 male probands, 20 (80%) and 5 (20%) were noncarriers and carriers respectively. All male mutation carriers had BRCA2 mutations. Table 1 shows the characteristics of the study population.

**Table 1:** Personal and family history of the probands (*N* = 310)

	BRCA					
	Negative		Positive		All	
	*n*	Col %	*n*	Col %	*n*	Col %
Female (*n* = 285)						
Personal history of breast cancer						
No	3	1.2	2	5.3	5	1.8
Yes	244	98.8	36	94.7	280	98.2
Personal history of ovarian cancer						
No	242	98.0	31	81.6	273	95.8
Yes	5	2.0	7	18.4	12	4.2
Personal history of breast and ovarian cancer						
No	245	99.2	33	86.8	278	97.5
Yes	2	0.8	5	13.2	7	2.5
Family history of breast cancer						
No	4	1.6	1	2.6	5	1.8
Yes	243	98.4	37	97.4	280	98.2
No. of family members with breast cancer (among those with family history with breast cancer)						
<3	223	91.8	21	56.8	244	87.1
≥3	20	8.2	16	43.2	36	12.9
Family history of ovarian cancer						
No	221	89.5	25	65.8	246	86.3
Yes	26	10.5	13	34.2	39	13.7
Family history of breast and ovarian cancer						
No	221	89.5	26	68.4	247	86.7
Yes	26	10.5	12	31.6	38	13.3
Male (*n* = 25)						
Personal history of breast cancer						
No	0	0	0	0	0	0
Yes	20	100	5	100	25	100
Family history of breast cancer						
No	0	0	0	0	0	0
Yes	20	100	5	100	25	100
No. of family members with breast cancer (among those with family history with breast cancer)						
<3	20	100	4	80	24	96
≥3	0	0	1	20	1	4
Family history of ovarian cancer						
No	20	100	5	100	25	100
Yes	0	0	0	0	0	0

Table 2 shows the mean and median predicted probabilities of mutation carriage for BRCA mutation carriers and noncarriers for female patients. We found that there was significant difference in mean predicted probability by all models for female BRCA carriers versus noncarriers. BRCA mutation carriers generally had higher mean scores than noncarriers. Table 3 shows that for males, none of the models showed any significant difference in mean predicted probability between BRCA carriers and BRCA noncarriers, although BRCA mutation carriers had higher model scores overall.

**Table 2:** Difference in scoring systems between BRCA-positive and BRCA-negative using two independent *t* statistics: females

	Mean	Median	Range	*t*	*p* value	95% CI
Female (*n* = 285)						
Age diagnosed with breast cancer (year)						
Noncarriers	44	44	18–82	1.48	0.14	(−0.92, 6.51)
Carriers	41.5	39	26–68			
All^a^	44	43.5	18–82			
Among carriers with breast cancer						
BRCA1	38	36	26–68	−1.75	**0.089** ^#^	(−12.35, 0.92)
BRCA2	44	41	28–63			
Age diagnosed with ovarian cancer (year)						
Noncarriers	34	31	19–50	−2.38	0.039*	(−30.23, −0.97)
Carriers	50	49	38–64			
All^b^	43.5	47.5	19–64			
Among carriers with ovarian cancer						
BRCA1	50	48	38–64	–	**–**	–
BRCA2	49	49	49			
BRCA 1						
Couch						
BRCA negative	10.55	7.7	0–77	−3.04	0.004*	(−19.58, −3.95)
BRCA positive	22.32	11.7	0–92.4			
All	12.12	7.7	0–92.4			
Shattuck-Eidens						
BRCA negative	7.11	4.2	0–74.8	−3.04	0.004*	(−16.71, −3.36)
BRCA positive	17.14	8.05	1.2–85.9			
All	8.44	4.6	0–85.9			
BRCAPRO						
BRCA negative	5.65	0.5	0–93.8	−4	<0.001*	(−33.41, −10.96)
BRCA positive	27.84	9.7	0–98.8			
All	8.61	0.8	0–98.8			
BOADICEA						
BRCA negative	4.37	1.17	0.03–90.07	−3.67	<0.001*	(−25.07, −7.26)
BRCA positive	20.53	7.75	0.24–99.41			
All	6.52	1.41	0.03–99.41			
BRCA 2						
BRCAPRO						
BRCA negative	4.55	1.2	0–61.4	−3.12	0.003*	(−18.32, −3.91)
BRCA positive	15.67	6.65	0–81.6			
All	6.04	1.6	0–81.6			
BOADICEA						
BRCA negative	3.62	1.72	0.07–39.1	−3.38	0.002*	(−11.71, −2.95)
BRCA positive	10.95	6.5	0.04–59.98			
All	4.59	1.82	0.04–59.98			
Any BRCA						
Myriad II						
BRCA negative	9.75	6.8	2.8–53.9	−3.66	0.001*	(−16.58, −4.79)
BRCA positive	20.43	15.8	2.9–79			
All	11.17	6.8	2.8–79			
BRCAPRO						
BRCA negative	10.19	2.5	0–99.2	−5.54	<0.001*	(−45.4, −21.12)
BRCA positive	43.45	42.35	0–100			
All	14.62	3.2	0–100			
BOADICEA						
BRCA negative	7.98	2.99	0.1–93.85	−4.34	<0.001*	(−34.45, −12.53)
BRCA positive	31.48	14.79	1.07–99.84			
All	11.12	3.4	0.1–99.84			

^a^There were five probands with ovarian cancer only, the number of patients with breast cancer is 280 (285-5)

^b^There were 12 probands in total with ovarian cancer (hence 7 with breast and ovarian cancers): 5 (41.7%) of them were noncarriers, 6 (50%) were BRCA1, and 1 (8.3%) was BRCA2

^#^ *p* < 0.1 (marginal significance); * *p* < 0.05

**Table 3:** Difference in scoring systems between BRCA-positive and BRCA-negative using two independent *t* statistics: males

	Mean	Median	Range	*t*	*p* value	95% CI
Male (*n* = 25)						
Age diagnosed with breast cancer (year)						
Noncarriers	62	64	33–83	0.8	0.431	(−8.3, 18.8)
Carriers (all BRCA2)	57	56	47–74			
All	61	63	33–83			
BRCA 1						
BRCAPRO						
BRCA negative	0.47	0	0–7.2	0.17	0.867	(−1.4, 1.64)
BRCA positive	0.34	0.3	0–0.8			
All	0.44	0	0–7.2			
BOADICEA						
BRCA negative	0.43	0.36	0.01–1.01	−1.34	0.192	(−0.45, 0.09)
BRCA positive	0.61	0.64	0.41–0.8			
All	0.47	0.41	0.01–1.01			
BRCA 2						
BRCAPRO						
BRCA negative	7.58	5.75	0–30.6	−1.37	0.241	(−69.88, 23.47)
BRCA positive	30.78	14.1	7.8–96.6			
All	12.22	7.8	0–96.6			
BOADICEA						
BRCA negative	6.74	7.22	0.03–15.77	−1.55	0.195	(−71.44, 20.05)
BRCA positive	32.43	15.5	8.61–95.84			
All	11.87	8.05	0.03–95.84			
Any BRCA						
Myriad II						
BRCA negative	12.75	12.8	2.8–21.8	−1.35	0.247	(−23.28, 7.94)
BRCA positive	20.42	12.8	12.8–41.9			
All	14.28	12.8	2.8–41.9			
BRCAPRO						
BRCA negative	8.04	5.75	0–30.8	−1.36	0.245	(−69.99, 23.82)
BRCA positive	31.12	14.1	7.8–97.2			
All	12.65	7.8	0–97.2			
BOADICEA						
BRCA negative	7.17	7.58	0.04–16.64	−1.56	0.193	(−71.67, 19.93)
BRCA positive	33.03	16.3	9.02–96.51			
All	12.33	8.64	0.04–96.51			

Observed and expected numbers of mutation carriers by predicted carrier probability using the models are shown in Tables 4, 5, and 6. BRCAPRO predictions are seen in Table 4. In females, 16 BRCA1 and 25 BRCA2 mutation carriers were predicted and 15 and 23 were observed respectively. For a total BRCA mutation prediction of 41 carriers when 38 were observed (*p* for goodness of fit = 0.91), this model performed the closest predicted carrier probability. In males, three, four, and three carriers were predicted using BRCAPRO, Myriad II, and BOADICEA models respectively and five were observed.

**Table 4:** Observed and expected number of mutation by predicted carrier probability under BRCAPRO: females and males

Carrier prob (%)			Observed						Expected			*p* value^†^
	No.	AC prob	No mutation		BRCA1		BRCA2		No mutation	BRCA1	BRCA2	
	*n*	%	*n*	row%	*n*	row%	*n*	row%	*n*	*n*	*n*	
Female												
<5	169	1.6	161	95.3	1	0.6	7	4.1	166.3	0.3	2.4	0.259
5 to <10	38	7.1	36	94.7	0	0	2	5.3	35.3	0.0	2.7	0.743
10 to <20	21	14.2	15	71.4	2	9.5	4	19	18	1	2	0.549
20 to <40	17	28.5	15	88.2	1	5.9	1	5.9	12.2	2.4	2.4	0.511
≥40	40	70.9	20	50	11	27.5	9	22.5	11.6	15.6	12.8	0.162
Total	285	14.6	247	86.7	15	5.3	23	8.1	243.4	16.4	25.2	0.91
Male												
<5	7	2.3	7	100	0	0	0	0	6.8	0	0.2	0.475
5 to <10	9	6.9	7	77.8	0	0	2	22.2	8.4	0	0.6	1.000
10 to <20	6	13.6	5	83.3	0	0	1	16.7	5.2	0	0.8	1.000
20 to <40	2	29.6	1	50	0	0	1	50	1.4	0	0.6	1.000
≥40	1	97.2	0	0	0	0	1	100	0	0	1	1.000
Total	25	12.7	20	80	0	0	5	20	21.8	0	3.2	0.702

*Carrier Prob* (%) range of carrier probability for each proband data; *No.* number of probands in the corresponding range; *AC Prob* (%) average carrier probability in the corresponding range

^†^Pearson χ^2^ goodness of fit test

**Table 5:** Observed and expected number of mutation by predicted carrier probability under Myriad II: *females and males*

Carrier prob (%)			Observed				Expected		*p* value^†^
	No.	AC Prob	No mutation		Any BRCA		No mutation	Any BRCA	
	*n*	%	*n*	row%	*n*	row%	*n*	*N*	
Female									
<5	46	2.9	45	97.8	1	2.2	44.7	1.3	1.000
5 to <10	161	6.7	148	91.9	13	8.1	150.2	10.8	0.671
10 to <20	45	15.8	35	77.8	10	22.2	37.9	7.1	0.419
20 to <40	27	33.8	16	59.3	11	40.7	17.9	9.1	0.573
≥40	6	58.4	3	50.0	3	50.0	2.5	3.5	1.000
Total	285	11.2	247	86.7	38	13.3	253.1	31.9	0.444
Male									
<5	1	2.8	1	100	0	0	0.97	0.03	1.000
5 to <10	0	0.0	0	0	0	0	0	0	–
10 to <20	21	12.8	18	85.7	3	14.3	18.3	2.7	1.000
20 to <40	2	21.8	1	50	1	50	1.6	0.4	1.000
≥40	1	41.9	0	0	1	100	0.6	0.4	1.000
Total	25	14.3	20	80	5	20	21.4	3.6	1.000

*Carrier Prob* (%) range of carrier probability for each proband data; *No.* number of probands in the corresponding range; *AC Prob* (%) average carrier probability in the corresponding range

^†^Pearson χ^2^ goodness of fit test

**Table 6:** Observed and expected number of mutation by predicted carrier probability under BOADICEA: *females and males*

Carrier prob (%)			Observed						Expected			*p* value^†^
	No.	AC Prob	No mutation		BRCA1		BRCA2		No mutation	BRCA1	BRCA2	
	*n*	%	*n*	row%	*n*	row%	*n*	row%	*n*	*n*	*n*	
Female												
<5	171	2.1	163	95.3	2	1.2	6	3.5	167.4	0.9	2.7	0.424
5 to <10	35	6.9	31	88.6	1	2.9	3	8.6	32.6	0.6	1.8	0.809
10 to <20	42	14.0	32	76.2	4	9.5	6	14.3	36.1	2.4	3.5	0.53
20 to <40	14	27.3	9	64.3	2	14.3	3	21.4	10.2	1.5	2.3	0.898
≥40	23	69.9	12	52.2	6	26.1	5	21.7	6.9	8.8	7.3	0.326
Total	285	11.1	247	86.7	15	5.3	23	8.1	253.4	12.5	19.2	0.723
Male												
<5	6	1.2	6	100.0	0	0.0	0	0.0	5.9	0.0	0.1	1.000
5 to <10	11	7.9	9	81.8	0	0.0	2	18.2	10.1	0.0	0.9	1.000
10 to <20	6	13.9	5	83.3	0	0.0	1	16.7	5.2	0.0	0.8	1.000
20 to <40	1	34.2	0	0.0	0	0.0	1	100.0	0.7	0.0	0.3	1.000
≥40	1	96.5	0	0.0	0	0.0	1	100.0	0.04	0.0	0.97	1.000
Total	25	12.3	20	80.0	0	0.0	5	20.0	21.9	0.0	3.1	0.702

*Carrier Prob (%)* range of carrier probability for each proband data; *No.* number of probands in the corresponding range; *AC Prob (%)* average carrier probability in the corresponding range

^†^Pearson χ^2^ goodness of fit test

As shown in Table 5, Myriad II predicted 32 female BRCA mutation carriers compared with the 38 observed (*p* = 0.444). For males, four were predicted and five were observed (*p* = 1.000). This was the best predictive model for males.

BOADICEA (Table 6) predicted 13 BRCA1 and 19 BRCA2 (32 in total) mutation carriers compared with 15 and 23 observed (38 in total) respectively (*p* = 0.723). In male probands, BOADICEA predicted three BRCA2 mutation carriers compared with five observed (*p* = 0.702).

For female probands, BRCAPRO (Table 4) tended to underestimate the number of BRCA1 and BRCA2 carriers at carrier probabilities <20%, but overestimated those ≥20%. Myriad II (Table 5) underestimated the number of BRCA1/2 mutation carriers for all carrier probabilities. BOADICEA (Table 6) underestimated the number of BRCA1 and BRCA2 carriers for carrier probabilities <40%, but overestimated those ≥40%. All models underestimated the expected number of males.

**Fig. 1:**
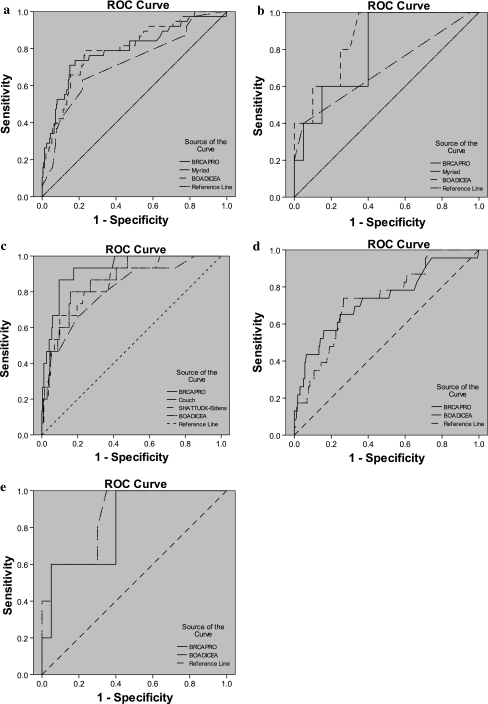
**a** ROC curves among difference models in female comparing BRCA carriers and noncarriers. Best model: BOADICEA, ROC = 0.8, *p* < 0.001. **b** ROC curves among difference models in male comparing BRCA carriers and noncarriers. Best model: BOADICEA, ROC = 0.87, *p* = 0.013. **c** ROC curves among difference models in female comparing BRCA1 and non-BRCA1 carriers. Best model: BRCAPRO, ROC = 0.93, *p* < 0.001. **d** ROC curves among difference models in female comparing BRCA2 and non-BRCA2 carriers. Best model: BOADICEA, ROC = 0.73, *p* < 0.001. **e** ROC curves among difference models in male comparing BRCA2 and non-BRCA2 carriers. Best model: BOADICEA, ROC = 0.87, *p* = 0.013

For BRCA1/2 prediction in females (Fig. 1a), the AUC was 0.79 using BRCAPRO, 0.72 using Myriad II, and 0.8 using BOADICEA. BRCAPRO had greatest AUC in BRCA1 specific (0.93) prediction compared with other models, and the same BRCA2 specific (0.73) prediction in females as BOADICEA. Conversely, the AUC was 0.8 using BOADICEA for BRCA1/2 mutation prediction in females and 0.87 in males (Fig. 1b); both were the highest scores compared with other models. Overall, BOADICEA had the highest discriminating power in females and males. Figures 1c and d show ROC curves for different models, comparing female BRCA1 carriers and noncarriers and BRCA2 carriers and noncarriers respectively. Figure 1e illustrates ROC curves for different models comparing male BRCA2 carriers and noncarriers.

Performance of BRCAPRO, Myriad II, Couch and Shattuck-Eidens, and BOADICEA models at conventional thresholds of 10% and 20% is shown in Table 7. In females, the highest sensitivity at both 10% and 20% cutoffs was achieved by BRCAPRO for BRCA1/2 mutations combined (73.7 and 57.9), BRCA1 mutations (86.7 and 66.7), and BRCA2 mutations (43.5 and 34.8), but its specificity was slightly lower than BOADICEA.

**Table 7:** Performance of BRCAPRO, Myriad II, Couch and Shattuck-Eidens, and BOADICEA at conventional thresholds of 10% and 20%

	Conventional threshold (%)	Sensitivity at conventional threshold (%)			Specificity at conventional threshold (%)		
		BRCA carrier vs. non-carrier	BRCA1 vs. non-BRCA1	BRCA2 vs. non-BRCA2	BRCA carrier vs. non-carrier	BRCA1 vs. non-BRCA1	BRCA2 vs. non-BRCA2
Female model							
BRCAPRO	10	73.7	86.7	43.5	79.8	86.7	90.1
	20	57.9	66.7	34.8	86.2	91.5	94.3
Myriad II	10	63.2	–	–	78.1	–	–
	20	34.2	–	–	92.3	–	–
Couch	10	–	80	–	–	64.8	–
	20	–	46.7	–	–	90.7	–
Shattuck-Eidens	10	–	66.7	–	–	80.4	–
	20	–	46.7	–	–	93	–
BOADICEA	10	68.4	60	30.4	78.9	89.3	90.8
	20	42.1	46.7	17.4	91.5	94.1	96.9
Male model							
BRCAPRO	10	60	–	60.0	75	–	75
	20	40	–	40.0	95	–	95
Myriad II	10	40	–	–	95	–	–
	20	20	–	–	100	–	–
BOADICEA	10	60	–	60	80	–	85
	20	40	–	40	100	–	100

In males, BOADICEA had similar sensitivity compared with BRCAPRO at both 10% and 20% cutoffs (60 and 40) in BRCA1/2 combined and BRCA2 (60 and 40) but a higher specificity for BRCA1/2 combined (80 and 100 vs. 750 and 95) and BRCA2 specificity (85 and 100 vs. 75 and 95). Myriad II generally had a lower sensitivity and specificity at both 10% and 20% cutoffs except for a slightly high specificity at 20% in BRCA1/2 combined in females (92.3). Couch and Shattuck-Eidens model had inferior sensitivities and specificities overall in our cohort.

## Discussion

Hong Kong, being the southern part of China, is a unique place to study hereditary breast cancers in Chinese with >80% of the study population originating from southern China [29, 36]. Moreover, the one-child policy in Mainland China is not practiced in Hong Kong, enabling larger family structures for analysis, which is relevant because limited familial history has been reported to result in underestimation of mutation carriers by various prediction models [33, 37]. In 2008, more than 2,600 new breast cancer cases were diagnosed (Hong Kong Cancer Registry) and it ranked the third most common cancer after lung and colorectal malignancies and the most common cancer in females. An improved understanding of hereditary breast cancer and more accurate selection of patients for genetic testing will have important implications for economic health policies.

A previous study performed by our group found 12.8% of clinically high-risk Chinese probands with breast and/or ovarian cancers carried a deleterious BRCA mutation, of which 60.7% were BRCA2 mutations [29]. This is a higher percentage of BRCA2 mutations compared with that of most Caucasian cohorts where studies have found that the prevalence of BRCA1 mutations ranges from 6.9% to 8.3% in European and American Caucasians compared with BRCA2 mutation prevalence of 5.2% to 5.9% [38–40], although consistent with other findings in Asian countries and a study performed by our group in Asian Americans [33, 41–43]. Mutations tend to be population-specific so different ethnic cohorts are likely to have a different spectrum of mutations and also different founder mutations [20, 44, 45]. In fact in a previous study, we reported a BRCA2 founder mutation in our cohort, which has accounted for the larger proportions of BRCA2 mutation carriers in our locality [36]. All of these differences are likely to result in inaccuracies in the use of existing prediction models, which have been designed based on Caucasian cohorts.

Asians comprise 57% of the world’s population and Chinese represent the largest group in the Asia continent [46], many of whom reside in western countries, such as the United States, where 4.2% of the population are Asian Americans. Therefore, Asian-specific studies and the accuracy of risk prediction models for use in this group would be of clinical relevance worldwide.

Our study indicated that BOADICEA is most accurate in predicting the numbers of BRCA1/2 mutation carriers combined compared with the other models with an AUC of 0.8 in females. It also had the closest predicted carrier probability for both male and female cohorts. Both BOADICEA and BRCAPRO models predicted the BRCA1 mutation carriage more accurately compared with the BRCA2 mutation carriage, although BRCA1 mutation carriage was better predicted by BRCAPRO: AUC was 0.87 (BOADICEA) and 0.93 (BRCAPRO), respectively.

Overall for the BRCAPRO and BOADICEA models, the AUC of 0.8 for BRCA1/2 mutations combined were higher than previous reports by Rao et al. (0.725) [34], Euhus et al. (0.712) [47], Marroni et al. (0.757) [48], Antoniou et al. (0.76) [49], and Kurian et al. (0.71 for Asians, 0.77 for whites) [33]. Possible reasons for predictions being higher in our study may be attributed to the differences in the prevalence of mutations, differences in mutation spectrum, and penetrance. Most other studies performed in Asians comprised a mixture of different Asian ethnic groups, including Vietnamese, Koreans, Filipinos, Malays, and Indians, whereas ours study was limited to southern Chinese. Moreover, a prediction model’s accuracy is dependent of the proband’s own account of family cancer history [50]. The reporting of family history may have differed in other studies where three generations of family history may not have been elicited which may also explain the differences in the estimation of BRCA mutations using these prediction models between studies [51]. Another possible explanation for the differences is the use of gender-specific analysis in our study. For the few males we included in our study, we found that the models did not have any different predictive probability between BRCA2 mutation carriers and noncarriers and they all underpredicted BRCA2 mutations by nearly twofold (there were no BRCA1 carriers). Risk prediction models should be used with caution in males because there is a lack of studies to assess the accuracy of the use of such models in male cohorts alone. One Caucasian study did find that BODICEA 5.0 can achieve a prediction sensitivity of 0.8 for BRCA1/2 and 0.63 for BRCA2 at 10% threshold [52]. A larger cohort of male probands is necessary to allow further confirmation of our studies’ findings. Consistent with previous reported studies, we found that both BOADCIEA and BRCAPRO models underestimated the number of mutations carriers at a lower threshold and overestimated at a higher threshold [53].

Recent studies have found that accuracy of risk prediction models can be improved by incorporating pathologic information into the algorithm [54]. Moreover, the use of risk-reduction strategies can affect the apparent penetrance of mutations and affect the prediction accuracy. Our previous studies found that approximately 20–30% of women with BRCA mutation elected for prophylactic contralateral mastectomy and salpingo-oophorectomy [55], including unaffected family members of probands who have found themselves to be mutation carriers. Revised versions of the BRCAPRO model incorporate such information [56] and may improve on the prediction accuracy that we report here.

The strength of this study is its representation of the broader Hong Kong population, because most cancer genetics referrals are seen at our institution through the referral to the Hong Kong Hereditary and High-Risk Breast Cancer Programme. Unlike many prior studies, complete genetic testing, including full gene sequencing and multiplex ligation-dependent probe amplifications (MLPA), was performed on all patients to minimize the chance of false negatives. Limitations include a clinic-based cohort rather than population-based setting, and a relatively small sample size especially for males, given the rarity of their disease.

We found underestimation of BRCA2 mutations in this Chinese cohort by standard mutation prediction models, despite relatively satisfactory discriminative ability; given that BRCA2 mutations are more prevalent in Asian and male cohorts, this finding has clinical significance. Further studies in larger cohorts, including Asians and males, are indicated, with the goal of developing an accurate predictive model specific to these populations and of targeting genetic testing more accurately for optimal patient care.
